# Impact of lipopolysaccharide-induced acute inflammation on baroreflex-controlled sympathetic arterial pressure regulation

**DOI:** 10.1371/journal.pone.0190830

**Published:** 2018-01-12

**Authors:** Takeshi Tohyama, Keita Saku, Toru Kawada, Takuya Kishi, Keimei Yoshida, Takuya Nishikawa, Hiroshi Mannoji, Kazuhiro Kamada, Kenji Sunagawa, Hiroyuki Tsutsui

**Affiliations:** 1 Department of Cardiovascular Medicine, Graduate School of Medical Sciences, Kyushu University, Fukuoka, Japan; 2 Department of Advanced Risk Stratification for Cardiovascular Diseases, Center for Disruptive Cardiovascular Medicine, Kyushu University, Fukuoka, Japan; 3 Department of Cardiovascular Dynamics, National Cerebral and Cardiovascular Center, Osaka, Japan; 4 Department of Therapeutic Regulation of Cardiovascular Homeostasis, Center for Disruptive Cardiovascular Medicine, Kyushu University, Fukuoka, Japan; Max Delbruck Centrum fur Molekulare Medizin Berlin Buch, GERMANY

## Abstract

**Background:**

Lipopolysaccharide (LPS) induces acute inflammation, activates sympathetic nerve activity (SNA) and alters hemodynamics. Since the arterial baroreflex is a negative feedback system to stabilize arterial pressure (AP), examining the arterial baroreflex function is a prerequisite to understanding complex hemodynamics under LPS challenge. We investigated the impact of LPS-induced acute inflammation on SNA and AP regulation by performing baroreflex open-loop analysis.

**Methods:**

Ten anesthetized Sprague-Dawley rats were used. Acute inflammation was induced by an intravenous injection of LPS (60 μg/kg). We isolated the carotid sinuses from the systemic circulation and controlled carotid sinus pressure (CSP) by a servo-controlled piston pump. We matched CSP to AP to establish the baroreflex closed-loop condition, whereas we decoupled CSP from AP to establish the baroreflex open-loop condition and changed CSP stepwise to evaluate the baroreflex open-loop function. We recorded splanchnic SNA and hemodynamic parameters under baroreflex open- and closed-loop conditions at baseline and at 60 and 120 min after LPS injection.

**Results:**

In the baroreflex closed-loop condition, SNA continued to increase after LPS injection, reaching three-fold the baseline value at 120 min (baseline: 94.7 ± 3.6 vs. 120 min: 283.9 ± 31.9 a.u.). In contrast, AP increased initially (until 75 min), then declined to the baseline level. In the baroreflex open-loop condition, LPS reset the neural arc (CSP-SNA relationship) upward to higher SNA, while shifted the peripheral arc (SNA-AP relationship) downward at 120 min after the injection. As a result, the operating point determined by the intersection between function curves of neural arc and peripheral arc showed marked sympatho-excitation without substantial changes in AP.

**Conclusions:**

LPS-induced acute inflammation markedly increased SNA via resetting of the baroreflex neural arc, and suppressed the peripheral arc. The balance between the augmented neural arc and suppressed peripheral arc determines SNA and hemodynamics in LPS-induced acute inflammation.

## Introduction

Intravenous injection of bacterial lipopolysaccharide (LPS) has been widely used as a model of acute endotoxemia [1–4]. Although LPS induces inflammatory responses in immune organs, its hemodynamic effects strikingly vary among studies. Ramchandra et al. [5] reported that an intravenous injection of *Escherichia coli* induces hypotension with increased cardiac output (CO) in sheep, which may mimic hyperdynamic circulation seen in human sepsis. In contrast, Forfia et al. [1] reported that LPS injection causes hypodynamic circulation with decreased CO in dogs. Radaelli et al. [2] demonstrated that arterial pressure (AP) can be maintained after LPS infusion in rats, despite increases in plasma concentrations of inflammatory cytokines. In order to understand complex and diverse hemodynamic changes after LPS challenge, we have to take various factors into considerations. Those include systemic vasodilation mediated by an increased production of nitric oxide [6], increased vascular permeability leading to a decrease in circulating plasma volume [7], reduction of vascular responsiveness to sympathetic nerve activity (SNA) [8], sympatho-excitation via direct activation of the central nervous system [9, 10], and variability of given LPS doses. In addition, arterial baroreflex may further modulate hemodynamic changes after LPS challenge [11]. The baroreflex may buffer changes in AP induced by the peripheral cardiovascular effect and in SNA induced by the central effect of LPS. Thus, examining the arterial baroreflex function is a prerequisite to understand hemodynamic changes induced by LPS.

One can evaluate the role of the arterial baroreflex on hemodynamics after LPS injection by comparing LPS effects on hemodynamics with and without baroreflex. Vayssettes-Courchay et al. [11] demonstrated that sinoaortic baroreceptor denervation accelerates a fall in AP after LPS treatment in rats, suggesting that the arterial baroreflex contributes to the maintenance of AP. Radaelli et al. [2] examined the effect of LPS on arterial baroreflex function by analyzing the baroreflex sensitivity; i.e., the relationship between AP and pulse interval, under baroreflex closed-loop condition. However, baroreflex sensitivity reflects the baroreflex-induced vagal response in heart rate and is incapable of evaluating the sympathetic baroreflex control of AP. Under the closed-loop condition, since AP is the input to the arterial baroreflex and at the same time the output from the baroreflex, the inevitable corruption of input and output variables makes it difficult to quantify the baroreflex control of AP in an unbiased manner. Furthermore, baroreflex sensitivity can be assessed only at AP around the operating point, which is a common drawback of closed-loop methods for the assessment of the baroreflex function [12].

To separate the baroreflex input (baroreceptor pressure) from the output (AP) and to evaluate the baroreflex function over the entire input range, we have employed open-loop analysis of the carotid sinus baroreflex [13, 14]. In this framework, we divided the carotid sinus baroreflex system into two principal subsystems. The neural arc subsystem characterizes the input-output relationship between carotid sinus pressure (CSP) and efferent SNA, whereas the peripheral arc subsystem characterizes the input-output relationship between efferent SNA and AP. Baroreflex equilibrium diagram enables us to understand how the operating point is determined through the interaction between the neural arc and peripheral arc[15, 16].

In this study, we investigated the impact of LPS-induced acute inflammation on SNA and AP regulation by performing baroreflex open-loop analysis in rats. We found that LPS-induced acute inflammation markedly increased SNA via resetting of the baroreflex neural arc, and suppressed cardiovascular responses to SNA (the peripheral arc).

## Materials and methods

### Animals and surgical preparations

Experiments and animal care were approved by the Committee on Ethics of Animal Experiment, Kyushu University Graduate School of Medical Sciences, and performed in strict accordance with the Guide for the Care and Use of Laboratory Animals released by the US National Institutes of Health.

We used 13 Sprague Dawley rats (weighing 380–583 g). A rat was anesthetized by intraperitoneal injection (2 ml/kg) of a mixture of α-chloralose (40 mg/ml) and urethane (250 mg/ml), and ventilated mechanically with oxygen-enriched air. The depth of anesthesia was maintained with a 15-fold diluted solution of the above anesthetic mixture infused from the right jugular vein (2–3 ml/kg/hour). Hence the infusion did not generate sizable pressure, we measured central venous pressure (CVP) from the same catheter using a fluid filled pressure transducer (model DX-200, Nihon Kohden, Tokyo, Japan). AP was measured using a 2 F high-fidelity pressure transducer (Model SPR-320, Millar Instruments; Houston, TX, USA) inserted into the left common carotid artery. Heart rate (HR) was detected from the arterial pressure waveform. A polyethylene tube (PE-50, Becton Dickinson, MD, USA) was inserted through the left femoral vein for drug injection. Through a right parasternal thoracotomy, a flow probe (Model MA2PSS; Transonic Systems, Ithaca, NY, USA) was attached to the aortic root for measurement of CO. Body temperature was maintained at approximately 37°C by a heating pad. A pair of stainless steel wire electrodes (Bioflex wire, AS632, Cooner Wire, CA, USA) was attached to a branch of the left splanchnic sympathetic nerve for SNA recording as described previously [15]. Preamplified nerve signals were band-pass filtered at 150–1000 Hz, and then full-wave rectified and low-pass filtered at a cutoff frequency of 30 Hz using analog circuits. At the end of the experiment, a bolus administration of a ganglionic blocker, hexamethonium bromide (60 mg/kg), was given to confirm the disappearance of SNA and to measure the noise level contaminated in the nerve signals.

### Carotid sinus baroreceptor isolation

**Fig 1:**
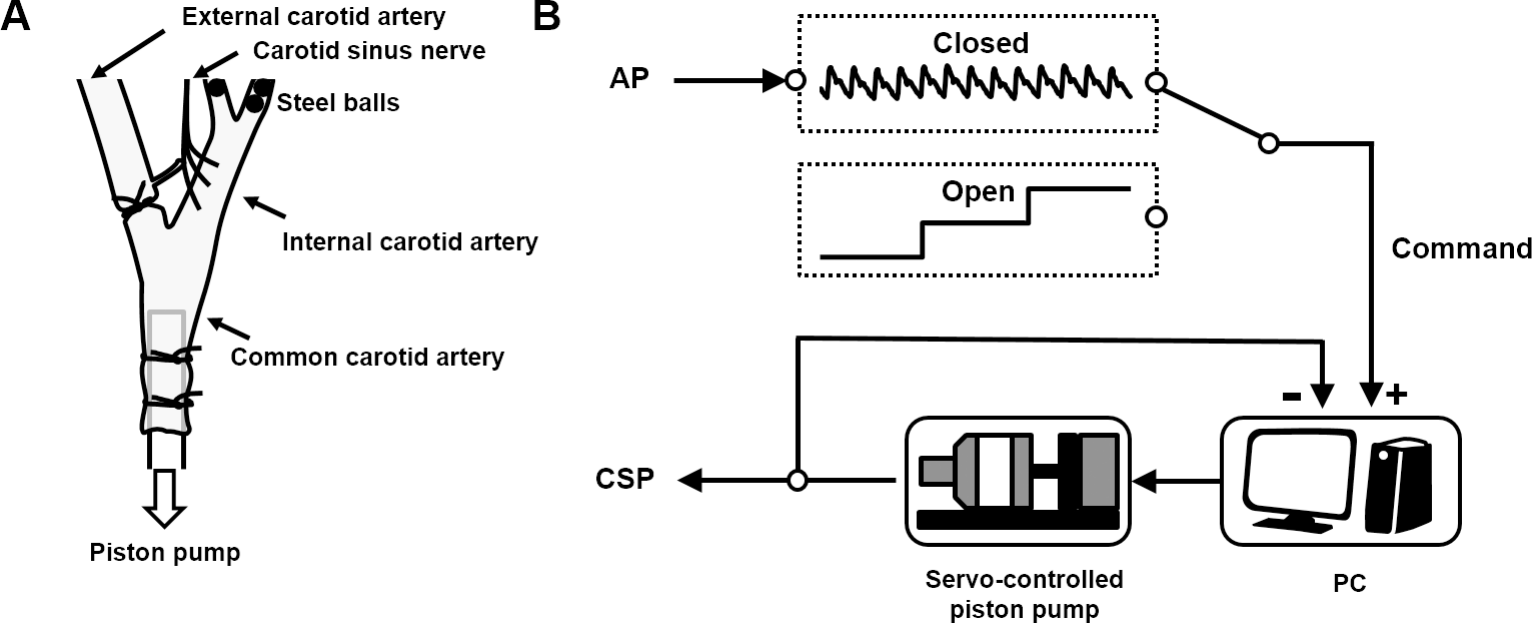
Scheme of the experiment. (A) Carotid sinus baroreceptor isolation. Bilateral external carotid arteries were ligated close to the carotid bifurcation and internal carotid arteries were embolized with steel balls. The isolated carotid sinuses were filled with physiological saline. The carotid sinus baroreceptor regions were connected to a pressure transducer and a servo-controlled piston pump. (B) Controlling carotid sinus pressure (CSP) by a servo-controlled piston pump. We matched CSP to AP in real time by the servo-controlled piston pump to establish the baroreflex closed-loop condition (Closed). We decoupled CSP from AP to establish the baroreflex open-loop condition (Open) and changed CSP stepwise (CSP ≠ AP) independent of AP to characterize baroreflex open-loop function.

Bilateral carotid sinus baroreceptor regions were isolated from the systemic circulation according to our previously reported procedures with modifications [17, 18]. Briefly, the external carotid artery was ligated close to the carotid bifurcation using a 7–0 silk thread. The internal carotid artery was embolized with five to seven steel balls (0.8 mm in diameter; Tsubaki Nakashima, Nara, Japan) injected from the common carotid artery. Vascular isolation was performed bilaterally, and the isolated carotid sinuses were filled with physiological saline through catheters inserted into the common carotid arteries (Fig 1A). Carotid sinus pressure (CSP) was controlled using a servo-controlled piston pump (Fig 1B). Bilateral aortic depressor nerves and vagal nerves were sectioned at the neck to minimize reflex effects from the aortic arch and the cardiopulmonary region.

### Induction of acute inflammation

Phenol-extracted LPS from *Escherichia coli* O55: B5 was purchased from Sigma-Aldrich (St Louis, MO, USA). LPS was diluted in saline to a concentration of 60 μg/ml and was sonicated for 20 min. LPS solution (1 ml/kg) was administered over a 5-min duration using a precision syringe pump (Legato^TM^ 110 Syringe Pump, KD Scientific Inc., PA, USA) via the left femoral intravenous catheter. The dose of LPS was based on a previous study [3].

### Assessment of serum TNF-α

The blood samples collected were centrifuged at 4°C and stored at -80°C for later assay. As an index of LPS-induced inflammation, the serum level of tumor necrosis factor-alpha (TNF-α) was determined using an ELISA kit (R&D Systems, MN, USA).

### Protocols

**Fig 2:**
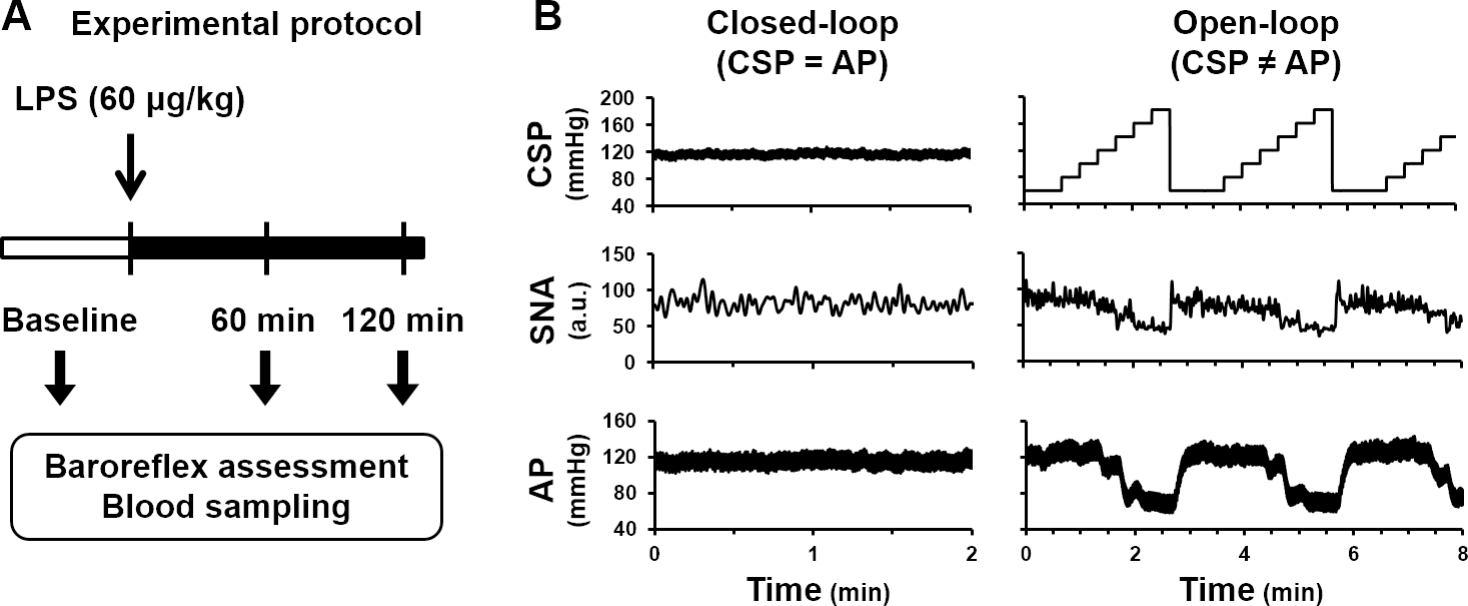
Experimental protocol. (A) We simultaneously recorded hemodynamics and sympathetic nerve activity (SNA) from baseline to 120 min every 15 min after lipopolysaccharide (LPS) injection. Baroreflex open-loop assessment and blood collection were performed at baseline and at 60 and 120 min after LPS injection. (B) Representative time series of carotid sinus pressure (CSP, mmHg), sympathetic nerve activity low-pass filtered at 0.3 Hz (SNA, a.u.) and arterial pressure (AP, mmHg) from one rat. In the baroreflex closed-loop condition, CSP was matched to AP. In the baroreflex open-loop condition, CSP was decoupled from AP and changed stepwise from 60 to 180 mmHg independent of AP to characterize the baroreflex function.

After the surgical procedures were completed, baroreflex responses to stepwise changes in CSP were monitored for more than 30 min. Rats were excluded if the reflex responses diminished during this stabilization period. We finally analyzed data from ten of the 13 rats. Fig 2A depicts the experimental protocol. After stabilization, we recorded baseline data and injected LPS (60 μg/kg) via the left femoral vein. To assess the impacts of LPS challenge on baroreflex function, we recorded SNA, AP, CO and CVP every 15 min for 120 min under the closed-loop condition. To evaluate the baroreflex open-loop characteristics, we temporarily opened the feedback loop at baseline, and at 60 min and 120 min after LPS injection. We changed CSP stepwise from 60 to 180 mmHg every 20 s for four cycles and measured changes in SNA and hemodynamics. Blood samples were collected from the left femoral artery (0.5 ml per sample, replenished with the same volume of physiological saline) at baseline, and at 60 and 120 min after LPS injection.

### Data analyses

Experimental data were recorded at 1000 Hz using a 16-bit analog-to-digital converter (Power Lab 16/35, AD Instruments, Sydney, Australia) and stored in a dedicated laboratory computer system. The noise level (determined as the average SNA signal after intravenous injection of 60 mg/kg hexamethonium bromide at the end of the experiment) was subtracted from recorded SNA. SNA was normalized by the level of SNA at CSP 60 mmHg at baseline. The data under the baroreflex closed-loop conditions were obtained every 15 min by averaging the time series for 60 s. Systemic vascular resistance (SVR) was calculated from: (AP-CVP)/CO.

For the baroreflex open-loop analysis, hemodynamic and SNA time series were averaged over the last 10-s at each CSP level. Static characteristics of the total baroreflex arc (CSP-AP relationship), neural arc (CSP-SNA relationship), HR response curve (CSP-HR relationship), and SVR response curve (CSP-SVR relationship) approximated an inverse sigmoid curve, and were quantified using a four-parameter logistic function as follows [19].

$$y=\frac{P_{1}}{1+exp[P_{2}(CSP-P_{3})]}+P_{4}$$

where y represents the output (AP, SNA, HR, or SVR); *P*_1_ is the response range of y; *P*_2_ is the slope coefficient; *P*_3_ is the midpoint of the sigmoid curve on the CSP axis; and *P*_4_ is the minimum value of y. The maximum gain is analytically derived as *P*_1_×*P*_2_/4 at CSP = *P*_3_. Static characteristics of the peripheral arc (SNA-AP relationship), the SNA-SVR relationship, and the SNA-CO relationship approximated a straight line, and were quantified using a linear regression as follows:

y=a×SNA+b

where y is AP, SVR, or CO; a and b represent the slope and intercept, respectively.

### Statistical analyses

Data are presented as means ± standard error of the mean (SEM) in each analysis. All data, except for the CSP-CO relationship, were evaluated by one-way factorial analysis of variance (ANOVA). Bonferroni test was used for post-hoc comparisons. For the CSP-CO relationship, differences were analyzed by a repeated-measures two-way (CSP and LPS) ANOVA. Tukey–Kramer test was used for post-hoc comparisons. Data were analyzed using statistical software (Social Survey Research Information Co., Ltd., Tokyo, Japan). Differences were considered significant when *p* < 0.05.

## Results

### Effect of LPS on sympathetic nerve activity and hemodynamics under baroreflex closed-loop condition

Serum TNF-α was undetectable under the baseline condition. LPS injection increased serum TNF-α to 1322.8 ± 240.1 pg/mL at 60 min. TNF-α declined to 92.9 ± 28.5 pg/mL at 120 min but remained above the detection limit.

**Fig 3:**
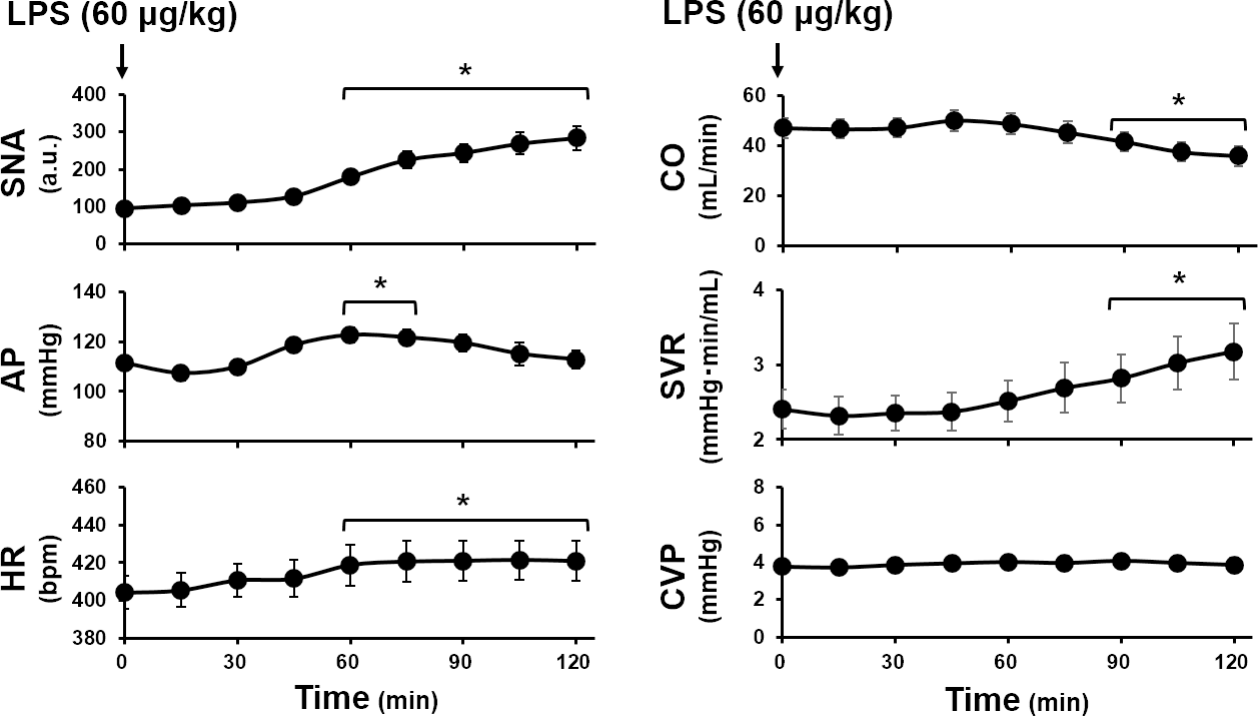
Changes in sympathetic nerve activity (SNA) and hemodynamics after lipopolysaccharide (LPS) injection under baroreflex closed-loop condition. Changes in SNA and hemodynamic parameters after LPS injection (60 μg/kg, iv) averaged over 10 rats. SNA, sympathetic nerve activity (normalized by baseline value); AP, arterial pressure (mmHg); HR, heart rate (bpm); CO, cardiac output (ml/min); SVR, systemic vascular resistance, (mmHg•min/ml); CVP, central venous pressure (mmHg). Data are expressed as mean ± SEM. * *p* < 0.05 vs. baseline.

Fig 3 shows the time courses of closed-loop SNA and hemodynamic measurements averaged over 10 rats. From 30 min after LPS injection, SNA increased continuously, reaching three-fold the baseline value at 120 min (baseline: 94.7 ± 3.6 vs. 120 min: 283.9 ± 31.9 a.u.). HR showed a trend similar to SNA. In contrast, AP increased gradually from 30 to 60 min after LPS injection, and was significantly higher than the baseline value at 60 and 75 min. Thereafter, AP declined toward the baseline value. CO was unchanged until 60 min after LPS injection, then decreased gradually and was significantly lower than the baseline value from 90 to 120 min. SVR nearly paralleled SNA and showed a trend opposite to CO. CVP did not change significantly throughout the observation period.

### Effects of LPS on baroreflex function

**Fig 4:**
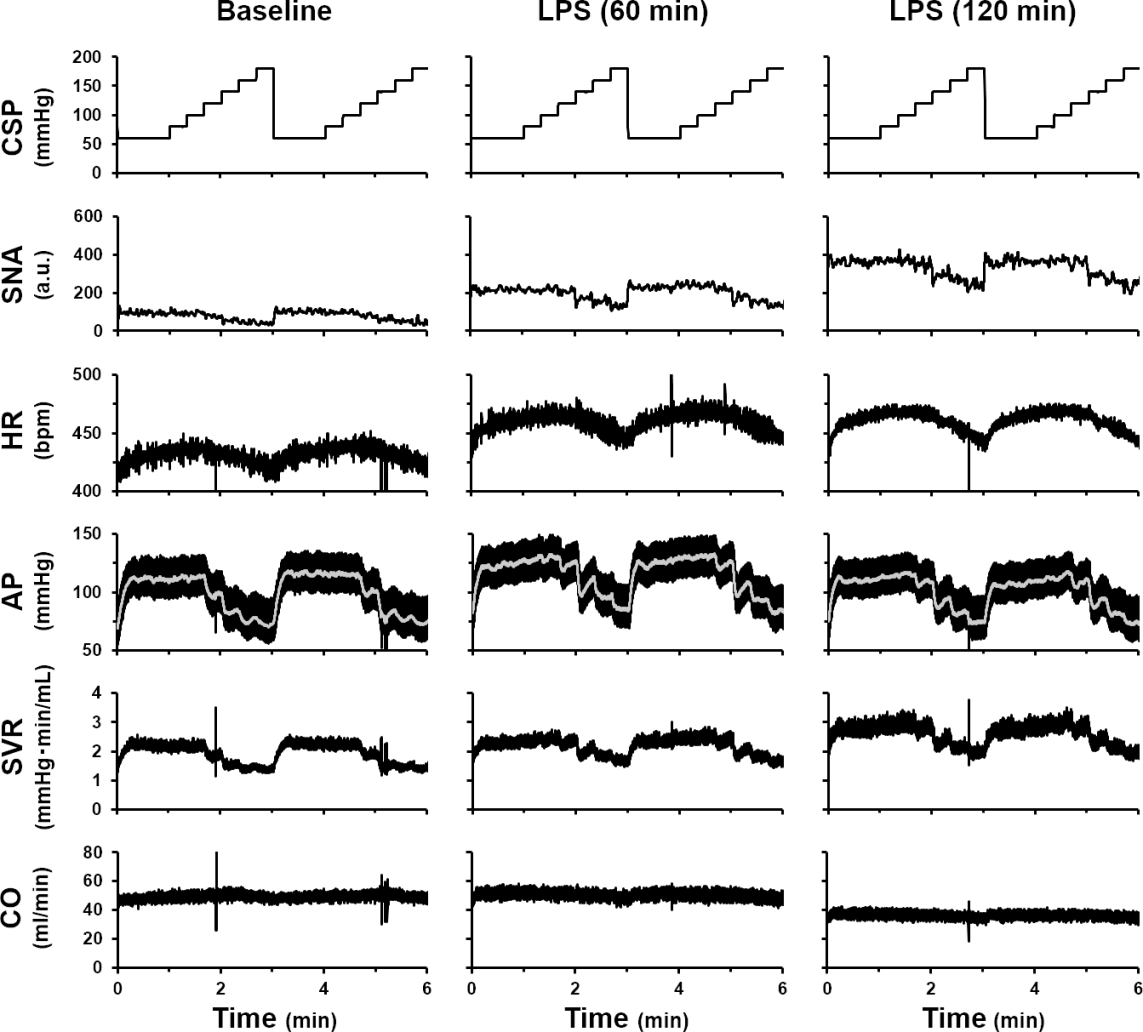
Typical time series of sympathetic nerve activity (SNA) and hemodynamics under baroreflex open-loop condition. Typical recordings of carotid sinus pressure (CSP), SNA, heart rate (HR), arterial pressure (AP), systemic vascular resistance (SVR), and cardiac output (CO) at baseline, and at 60 and 120 min after LPS injection. Gray lines in the panels of AP indicate 1-s moving averaged data.

Illustrated in Fig 4 are typical recordings of SNA and hemodynamics obtained at baseline, and at 60 and 120 min after LPS injection. At baseline, an increase in CSP decreased SNA, HR, AP, and SVR. While maintaining these relationships, SNA, HR, and SVR increased with time after LPS injection. In contrast AP increased at 60 min and then decreased at 120 min. Although changes in CSP little affected CO throughout this experiment, CO decreased significantly at 120 min.

**Fig 5:**
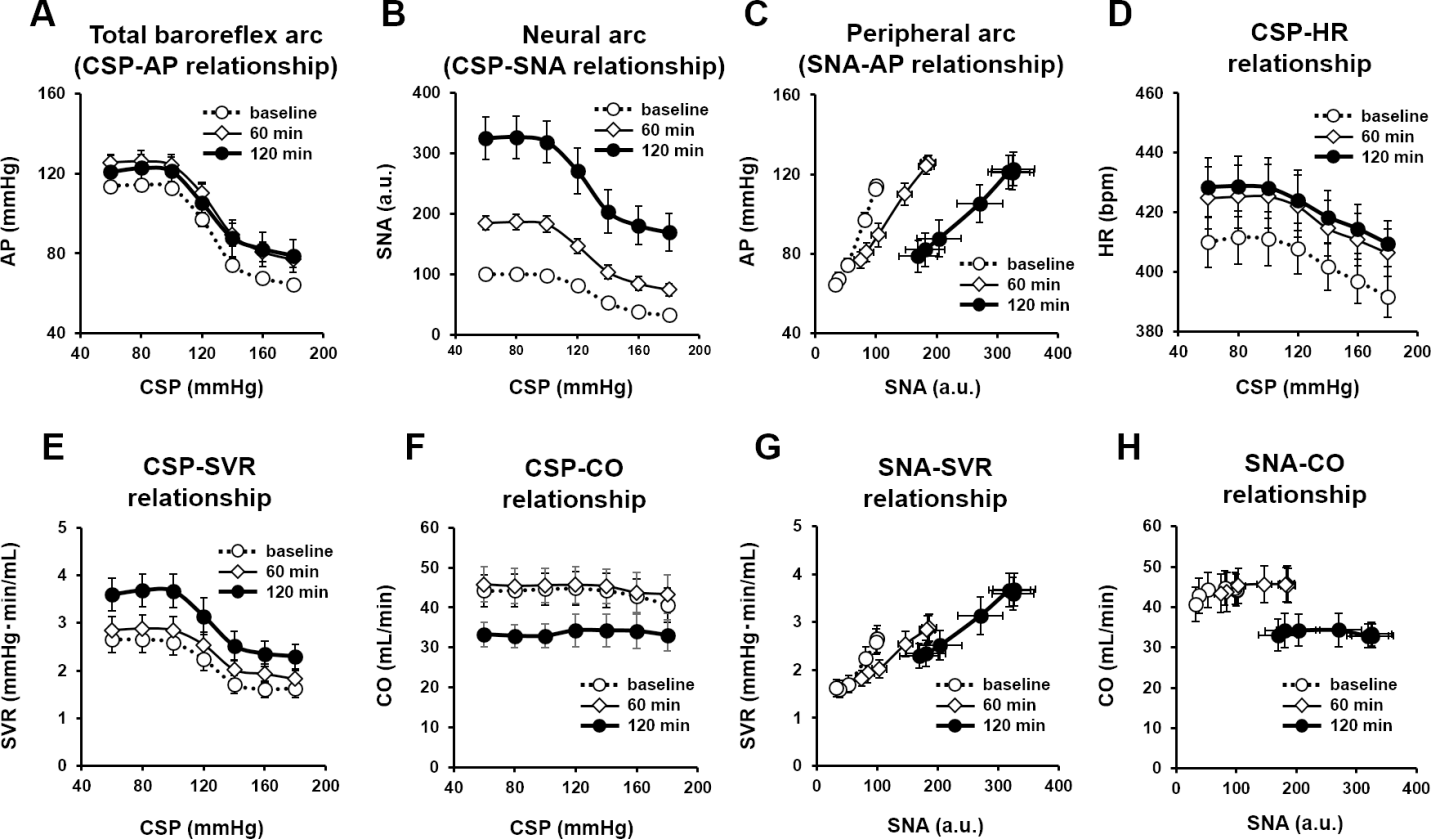
Open-loop characteristics of the baroreflex under LPS. Open-loop static characteristics of the total baroreflex arc (A), neural arc (B), peripheral arc (C), CSP to HR relationship (D), CSP to SVR relationship (E), CSP to CO relationship (F), SNA to SVR relationship (G), and SNA to CO relationship (H) obtained at baseline (dotted line with white circles, ○), and 60 min (thin solid line with diamonds, ◇) and 120 min after LPS (thick solid line with black circles, ●). Data are expressed as means ± SEM (n = 10). CSP, carotid sinus pressure (mmHg); AP, arterial pressure (mmHg); SNA, sympathetic nerve activity (a.u.); HR, heart rate (bpm); SVR, systemic vascular resistance (mmHg•min/ml); CO, cardiac output (ml/min).

Fig 5 summarizes the open-loop static characteristics of baroreflex under the baseline condition, and at 60 and 120 min after LPS injection obtained from 10 rats. For the total baroreflex arc (Fig 5A), increases in CSP decreased AP sigmoidally. LPS marginally moved the total baroreflex arc toward higher AP reflecting a significant increase in minimum AP at 120 min compared to baseline (Table 1). LPS had no significant effects on the range of AP response, midpoint input pressure, or maximum gain of the total baroreflex arc. The baroreflex neural arc showed an inverse sigmoidal curve (Fig 5B). LPS increased the range of SNA response and minimum SNA in a time-dependent manner (Table 1). Maximum gain of the neural arc was significantly higher at 120 min compared to baseline. For the baroreflex peripheral arc, increases in SNA increased AP linearly (Fig 5C). LPS decreased the slope of the regression line in a time-dependent manner, but did not affect the intercept values of AP (Table 1). LPS increased minimum HR at 60 min and 120 min compared to baseline, without affecting other parameters (Fig 5D and Table 1). LPS did not affect the CSP-SVR relationship significantly at 60 min, but moved the curve upward at 120 min (Fig 5E and Table 1). The effects of CSP and LPS on CO were examined by two-way ANOVA followed by Tukey-Kramer test (Fig 5F). CSP did not affect CO significantly. In contrast, LPS reduced CO significantly at 120 min. There was no apparent interaction between the effects of CSP and LPS on CO. For the SNA-SVR relationship (Fig 5G and Table 1), LPS decreased the slope at 60 and 120 min significantly compared to baseline. However, unlike the peripheral arc, the difference of the slope between 60 min and 120 min was not significant. The slope of the SNA-CO relationship did not differ among the three conditions, while LPS decreased the intercept significantly at 120 min (Fig 5H and Table 1).

**Table 1:** Parameters of open-loop characteristics of the baroreflex.

	Baseline			LPS (60 min)			LPS (120 min)		
**CSP-AP (Total baroreflex arc)**									
*P*_1_, mmHg	49.6	±	3.7	50.2	±	3.1	42.4	±	4.4
*P*_2_, mmHg/mmHg	0.123	±	0.012	0.119	±	0.019	0.149	±	0.020
*P*_3_, mmHg	126.7	±	2.3	128.4	±	4.2	126.4	±	3.1
*P*_4_, mmHg	65.1	±	2.7	77.2	±	3.6	79.9	±	7.9 *
G_max_, mmHg/mmHg	1.52	±	0.18	1.55	±	0.28	1.63	±	0.33
**CSP-SNA (Neural arc)**									
*P*_1_, a.u.	68.7	±	4.5	114.9	±	9.1 *	156.8	±	22.0 *^†^
*P*_2_, a.u./mmHg	0.101	±	0.008	0.113	±	0.019	0.139	±	0.019 *
*P*_3_, mmHg	130.9	±	2.2	128.1	±	3.5	126.3	±	3.1
*P*_4_, a.u.	32.4	±	4.4	73.9	±	9.3 *	170.1	±	31.3 *^†^
G_max_, a.u./mmHg	1.72	±	0.15	3.35	±	0.71	5.36	±	1.01 *^†^
**SNA-AP (Peripheral arc)**									
Slope, mmHg/a.u.	0.748	±	0.040	0.452	±	0.035 *	0.290	±	0.028 *^†^
Intercept, mmHg	37.9	±	5.0	44.5	±	6.7	34.8	±	8.4
**CSP-HR**									
*P*_1_, bpm	23.6	±	3.3	19.5	±	3.5	20.5	±	3.1
*P*_2_, bpm/mmHg	0.077	±	0.005	0.081	±	0.003	0.079	±	0.006
*P*_3_, mmHg	142.7	±	2.7	139.7	±	2.9	138.9	±	2.2
*P*_4_, bpm	385.9	±	7.0	401.1	±	7.4 *	401.3	±	7.8 *
G_max_, bpm/mmHg	0.43	±	0.04	0.39	±	0.07	0.38	±	0.05
**CSP-SVR**									
*P*_1_, mmHg•min/mL	1.10	±	0.15	1.15	±	0.16	1.46	±	0.21
*P*_2_, min/mL	0.14	±	0.02	0.12	±	0.02	0.13	±	0.02
*P*_3_, mmHg	123.6	±	2.6	127.3	±	4.5	126.3	±	3.2
*P*_4_, mmHg•min/mL	1.58	±	0.18	1.79	±	0.17	2.26	±	0.26 *^†^
G_max_, mL/min	0.033	±	0.007	0.032	±	0.008	0.047	±	0.010
**SNA-SVR**									
Slope, mmHg•min/mL /a.u.	0.017	±	0.002	0.010	±	0.001 *	0.009	±	0.001 *
Intercept, mmHg•min/mL	0.92	±	0.11	1.08	±	0.14	0.97	±	0.22
**SNA-CO**									
Slope, mL/min/a.u.	0.025	±	0.018	0.018	±	0.013	-0.002	±	0.009
Intercept, mL/min	44.3	±	3.7	45.2	±	4.2	36.6	±	4.6 *^†^

Data are expressed as means ± SEM (n = 10). CSP, carotid sinus pressure; AP, arterial pressure; SNA, sympathetic nerve activity; HR, heart rate; SVR, systemic vascular resistance; CO, cardiac output; *P*_1_ to *P*_4_, parameters of a 4-parameter logistic function; *P*_1_, response range; *P*_2_, coefficient of gain; *P*_3_, midpoint of the operating range; *P*_4_, minimum value; G_max_, maximum gain calculated from *P*_1_× *P*_2_ / 4. Slopes and intercepts are calculated from linear regression.

* *p <* 0.05 vs. baseline

† *p* < 0.05 vs. LPS (60 min).

**Fig 6:**
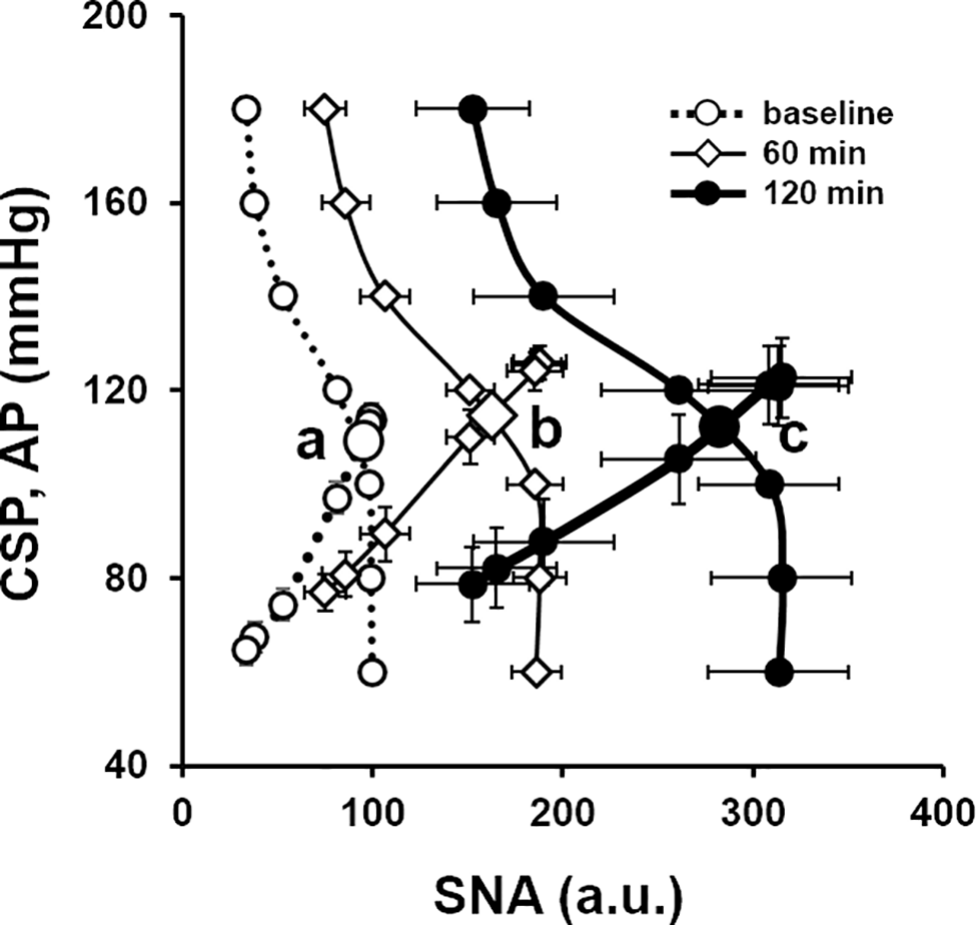
Baroreflex equilibrium diagram under LPS. Averaged baroreflex equilibrium diagram at baseline (dotted line with white circle, operating point *a*), and at 60 min (thin solid line with diamond, operating point *b*) and 120 min after LPS injection (thick solid line with black circle, operating point *c*). SNA, sympathetic nerve activity (a.u.); CSP, carotid sinus pressure (mmHg); AP, arterial pressure (mmHg).

Fig 6 shows the baroreflex equilibrium diagram of averaged neural arc and peripheral arc under baseline conditions, and at 60 and 120 min after LPS injection. The intersections between the neural arc and peripheral arc represent the operating points. The operating point SNA was 92.6 ± 1.76 a.u. at baseline (point *a*), and increased significantly to 160.2 ± 11.4 a.u. at 60 min (point *b*) and 281.8 ± 28.4, a.u. at 120 min (point *c*) after LPS injection. The operating point AP increased slightly at 60 min after LPS injection and returned toward the baseline level at 120 min. Despite striking increases in SNA, the operating point AP remains remarkably constant.

## Discussion

The major findings of this study are as follows. LPS-induced acute inflammation activated SNA markedly by resetting the baroreflex neural arc, while shifting the peripheral arc downward. In addition, LPS attenuated the SVR response to SNA and decreased CO without changing CVP. As a result of augmentation of neural arc and suppression of peripheral arc, the operating point, which is the intersection between the function curves of neural arc and peripheral arc, showed marked sympatho-excitation without substantial changes in AP.

### Effects of LPS on hemodynamics under baroreflex closed-loop conditions

LPS is the outer cell envelope of Gram negative bacteria. Upon entering blood stream, LPS causes endotoxemia with systemic inflammation. TNF-α, an inflammatory cytokine, was not detectable under baseline conditions, but increased significantly at 60 min after LPS injection, confirming the LPS-induced acute inflammatory response. Although endotoxemia can cause hypotension with or without increases in CO in large animals [1, 20], hypotension was not observed at the dose of LPS (60 μg/kg) used in the present study (Fig 3). Vayssettes-Courchay et al. [11] demonstrated that a high dose of LPS (20 mg/kg) caused hypotension in rats, but AP remained above 90 mmHg for 120 min after LPS injection. These previous studies and our results indicate that the impact of LPS on AP varies depending on the LPS dose and experimental conditions.

Although LPS has been known to induce powerful vasodilatation [21], LPS increased SVR in this study (Fig 3). The fact that SVR paralleled SNA suggests that concomitant increases in SNA may account for the increase in SVR. We will discuss this in detail in the next section with respect to the SNA-SVR relationship (Fig 5G). Furthermore, LPS did not induce shock hemodynamics contrary to previous reports [1, 20]. Since the product of SVR and CO determines AP, increases in SVR without decreases in CO inevitably increased AP from 60 to 75 min after LPS injection. Thereafter, even though SVR continued to increase, the overwhelming decrease in CO decreased AP toward the baseline level at 120 min. We will discuss the mechanism of CO alterations on the basis of the SNA-CO relationship (Fig 5H) and Guyton’s circulatory equilibrium in the next section.

### Effects of LPS on open-loop characteristics of the baroreflex

As shown in Fig 5B, LPS reset the neural arc markedly upward. Previous studies indicate that LPS increases pro-inflammatory cytokines in intracerebral tissue, leading to sympatho-excitation [10, 22]. LPS also directly induces sympatho-excitation via Toll-like receptor 4 activation or endoplasmic reticulum stress in paraventricular nuclei [4]. Two opposing interpretations are possible regarding the upward resetting of the neural arc, as discussed below. Focusing on the absolute level of sympathetic suppression, one can interpret that the LPS-induced sympatho-excitation reduces the baroreflex function because LPS increases minimum SNA (Table 1). However, the LPS-induced sympatho-excitation is more pronounced at low CSP levels than at high CSP levels (Fig 5B). Therefore, if one focuses on the magnitude of sympathetic suppression, LPS widens the response range of SNA and increases the maximum gain of the baroreflex neural arc (Table 1).

Because the neural arc showed an upward resetting with an increase in maximum gain, LPS would have markedly elevated AP if LPS had not suppressed the peripheral arc characteristics. However, the total baroreflex arc showed only a slight upward resetting without significant changes in maximum gain (Fig 5A), due to the attenuation of AP response to SNA (Fig 5C). Previous studies have indicated that high-dose LPS (10 mg/kg, i.v.) decreases the baroreflex function [4], whereas low-dose LPS (10 μg/kg, i.v.) enhances it [23]. We speculate that the balance between changes in neural arc and peripheral arc determines the baroreflex function in response to LPS challenge.

LPS decreased the slope of the peripheral arc in a time-dependent manner (Table 1). We further divided the peripheral arc into the SNA-SVR and SNA-CO relationships (Fig 5G and 5H). LPS has been shown to induce vascular dilation by inducing nitric oxide and cyclic guanosine monophosphate production [24]. The vasodilatory effect was not evident from the CSP-SVR relationship, which showed higher SVR at any given CSP after LPS injection (Fig 5E). However, LPS injection significantly decreased the slope of the SNA-SVR relationship at 60 and 120 min (Fig 5G). Hence the LPS induced increase in SNA overrode the reduction of SVR responsiveness, leading to an apparent increase in SVR for any given CSP.

As shown in Fig 5H, LPS shift SNA-CO relationship downward with higher SNA at 120 min. We further interpreted those changes in CO by Guyton's circulatory equilibrium framework. In the framework, the intersection between the CO curve and venous return curve [25] determines CO. In the baroreflex closed-loop condition, CO remained unchanged until 90 min after LPS injection, and decreased thereafter from 90 to 120 min without changing CVP (Fig 3). We speculate that these hemodynamic changes may indicate downward shifts of the CO curve and venous return curve (see S1 Fig). It is well known that elevations of SVR flatten the CO curve. In contrast, increases in HR and cardiac contractility steepen the CO curve. HR was increased at 120 min after LPS injection. We did not directly evaluate cardiac contractility in this study. However, it is conceivable that excessive sympatho-excitation may have increased cardiac contractility similar to the SVR response to LPS. On the contrary, both LPS and LPS-induced inflammatory cytokines are known to worsen cardiac contractility [1, 24]. The net effect of LPS on cardiac contractility remains to be clarified. Nevertheless, since LPS reduced CO and accelerated HR without changing CVP at 120 min after injection, the impact of LPS on SVR appears to override that on HR and cardiac contractility in determining CO.

Regarding the venous return, LPS reduced CO at 120 min after injection without changing CVP. This is possible only when stressed volume is reduced. If stressed volume remains unchanged, the decrease in CO should increase CVP. LPS induced venodilation, a parallel pooling of blood in abdomen and large amounts of volume shifting from the intravascular space to the interstitial space [7] would have shifted the venous return curve downward. On the other hand, the baroreflex mediated or non-mediated, e.g. a direct LPS effect, sympatho-excitation may induce venoconstriction through α adrenoreceptors and increase stressed blood volume [26]. The results of this study indicated that the net effect of those antagonizing impacts of LPS on stressed volume was the reduction of stressed volume. As a result, the venous return curve shifted downward at 120 min after LPS injection.

As for the impact of LPS on baroreflex controlled HR, LPS time-dependently reset the relationship upward (Fig 5D). In addition, we also evaluated the SNA-HR relationship (S2 Fig). As can be seen, the relationship remains hardly changed between baseline and 60 min after LPS, despite the fact that the operating range of SNA nearly doubled. In contrast, 120 min after LPS, the operating range of SNA further increased between 180 to 320%. However, the SNA and HR relationship clearly shifted downward. In other words, for the same level of SNA, HR was lower 120 min after LPS compare to earlier periods. Zhou et al. reported that the endotoxin induced sympatho-adrenal activation increases plasma catecholamine level independent of the baroreflex [27], thereby increases HR. In addition, Takayama et al. reported that inflammatory tachycardia is caused by a direct action on the heart of thromboxane A2 and prostaglandin F2α [28]. On the other hand, Zorn-Pauly et al. reported that LPS reduced If current, leading to a longer cycle length in the sinoatrial node [29]. How those various mechanisms are responsible for the downward shift of the SNA and HR relationship 120 after LPS injection remains to be clarified.

### Baroreflex equilibrium diagram

**Fig 7:**
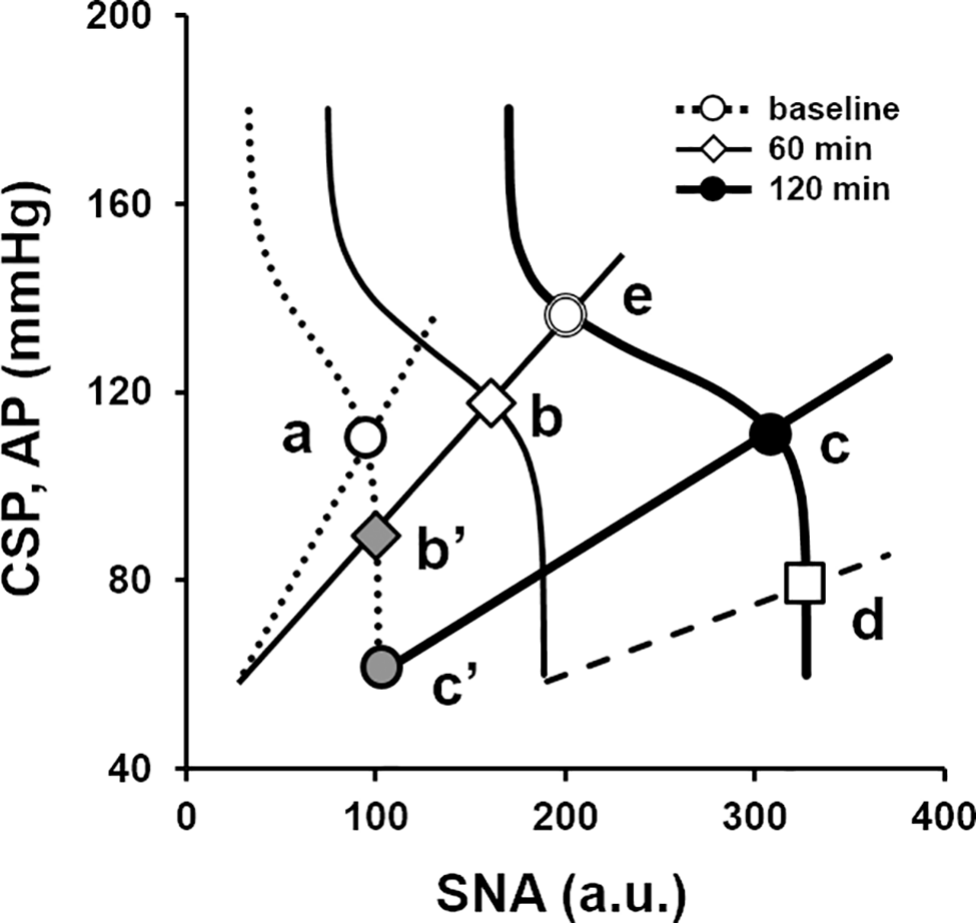
Baroreflex equilibrium diagram under LPS and septic shock. Baroreflex equilibrium diagram drawn from averaged parameters at baseline (dotted line with white circle, operating point *a*), and at 60 min (thin solid line with diamond, operating point *b*) and 120 min after LPS injection (thick solid line with black circle, operating point *c*). Without neural arc resetting, LPS would move the operating point from *b* to *b'* at 60 min and *c* to *c'* at 120 min, causing severe hypotension. In the case of septic shock, the marked downward shift in the peripheral arc (dashed line) induces catecholamine refractory hypotension (operating point *d*). If we assume that the shift in the peripheral arc from 60 min to 120 min results from the contraction of stressed volume, replenishing the stressed volume restores the peripheral arc at 60 min and the operating point approaches *e*. SNA, sympathetic nerve activity (a.u.); CSP, carotid sinus pressure (mmHg); AP, arterial pressure (mmHg).

The baroreflex equilibrium diagram provides mechanistic insights on how AP and SNA at the operating point are determined and helps understand the baroreflex function under pathophysiological conditions [15, 16]. As schematized in Fig 7, LPS injection resets the neural arc toward higher SNA and decreases the slope of the peripheral arc. After LPS challenge, despite the marked increase in operating point SNA, the operating point AP did not increase much at 60 min (*b*) or 120 min (*c*) after LPS injection. If the neural arc had not reset after LPS challenge, the operating point would have moved to *b'* at 60 min and *c'* at 120 min, causing severe hypotension. In other words, the balance between changes in neural and peripheral arcs determines AP and SNA after LPS challenge.

High-dose LPS induces severe hypotension with sympatho-excitation in rats [11]. In addition, LPS challenge has been used as a model of septic shock, in which acute inflammation induces hemodynamic collapse [30]. The baroreflex equilibrium diagram illustrates this pathophysiology as a further downward shift of the peripheral arc, which moves the operating point from *c* to *d*. This explains the profound hypotension and excessive sympatho-excitation under this pathophysiological condition.

The downward shift of the peripheral arc from 60 min to 120 min after LPS injection can be explained by the reduction of stressed volume as discussed earlier. Therefore, replenishing the stressed volume would restore the peripheral arc at 60 min, increase AP and CO, and markedly decrease SNA (point e) at least for the short term. However, severe inflammation would alter cardiovascular properties progressively. The worsening of cardiac contractility shifts the CO curve downward. In addition, further decreases in stressed volume may shift the venous return curve downward even more. In those settings, volume restoration alone may not be capable of normalizing hemodynamics. Thus, the treatment targeting both hemodynamic and fundamental pathophysiology of septic shock should be conducted.

### Limitations

There are several limitations in this study. First, we performed the experiment in animals under anesthetized conditions. Since anesthesia is known to reduce baroreflex function as well as SNA, the results of the present study may not be directly applicable to a conscious state or clinical setting. Second, the study examined the interaction between acute inflammation and baroreflex controlled SNA. It is well known that the link between chronic inflammation and SNA plays a pivotal role in the pathogenesis of several cardiovascular diseases [31] and autoimmune disorder [32]. It remains unknown whether the present results can be extrapolated to such situations. Third, we cut the vagal nerves to allow open-loop analysis of the carotid sinus baroreflex. The vagal nerves have been shown to play an important role in the inflammatory response. As an example, Goehler et al. [33] reported that vagal nerves delivered signals of systemic inflammatory information to the central nervous system. Tracy et al. [34] reported that vagal nerves reduced the inflammatory response via the cholinergic anti-inflammatory pathway. Contrary to this, Martelli et al. [3] reported that inflammatory responses (plasma TNF-α and corticosterone levels), which were induced by the same low dose of LPS used in this study, did not change significantly with or without vagal nerve in rats. Further investigations are needed to establish the effect of vagal nerves on inflammatory-baroreflex interaction. Lastly, we did not include a control group in the present study. There is a possibility that the observed changes that we interpreted as the effects of LPS injection could be LPS-independent, time dependent changes in baroreflex function and hemodynamics. However, our preliminary study indicated that baroreflex function and hemodynamics remained stable for at least 2 hours after vehicle injection (see S3 Fig). Therefore, we consider the results of this study as the effects of LPS injection.

## Conclusions

In conclusion, LPS-induced inflammation markedly increased SNA via resetting of the baroreflex neural arc, and decreased the cardiovascular response to SNA. Separate assessments of the characteristics of neural arc and peripheral arc are crucial to understand the complex hemodynamic changes after LPS challenge.

## Supporting information

S1 Fig Graphical representation of hypothetical circulatory equilibrium in baseline and 120 min after LPS. Thin and thick solid lines indicate the cardiac output curves in baseline and 120 min after LPS, respectively. Thin and thick dashed lines indicate the venous return curves in baseline and 120 min after LPS, respectively. Open (○) and closed (●) circles represent the equilibrium point in baseline and 120 min after LPS, respectively. Since we did not measure the mean systemic filling pressure which is essential to characterize circulatory equilibrium, two lines crossing at the operating point before and after LPS are hypothetical cardiac output curve and venous return curve, respectively.
(TIF)

S2 Fig Changes in SNA and HR relationship after LPS injection. Open-loop static characteristics of sympathetic nerve activity (SNA) and heart rate (HR) obtained at baseline (dotted line with white circles, ○), and 60 min (thin solid line with diamonds, ◇) and 120 min after Lipopolysaccharide (LPS) injection (thick solid line with black circles, ●). Data are expressed as means ± SEM. Data are acquired from the baroreflex open loop condition at baseline and at 60 and 120 min after LPS injection.
(TIF)

S3 Fig Changes in baroreflex equilibrium diagram after vehicle infusion. We used saline as vehicle. Methods and protocols for obtaining the baroreflex equilibrium diagram are described in the method section. Averaged baroreflex equilibrium diagrams at baseline (dotted line with white circle, ○), 60 min (thin solid line with diamond, ◇) and 120 min after vehicle injection (thick solid line with black circle, ●) are shown. We did not observe significant changes in the characteristics of central arc or peripheral arc at least until 120 min. Each data was obtained from three rats.
(TIF)

## Abbreviations

AP: arterial pressure
CO: cardiac output
CSP: carotid sinus pressure
CVP: central venous pressure
HR: heart rate
LPS: lipopolysaccharide
SNA: sympathetic nerve activity
SVR: systemic vascular resistance
TNF-α: tumor necrosis factor-alpha
