# Supplementary figures and images for: Impact of lipopolysaccharide-induced acute inflammation on baroreflex-controlled sympathetic arterial pressure regulation
Source: PLoS One. 2018 Jan 12;13(1):e0190830. doi: 10.1371/journal.pone.0190830 (PMC5766102; doi:10.1371/journal.pone.0190830)

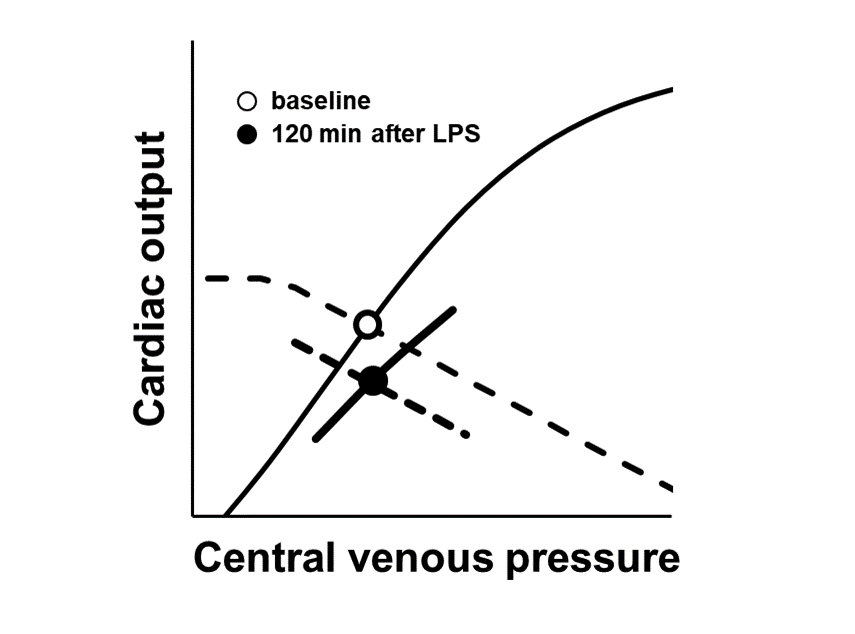

Supplement: S1 Fig — Thin and thick solid lines indicate the cardiac output curves in baseline and 120 min after LPS, respectively. Thin and thick dashed lines indicate the venous return curves in baseline and 120 min after LPS, respectively. Open (○) and closed (●) circles represent the equilibrium point in baseline and 120 min after LPS, respectively. Since we did not measure the mean systemic filling pressure which is essential to characterize circulatory equilibrium, two lines crossing at the operating point before and after LPS are hypothetical cardiac output curve and venous return curve, respectively. (TIF) [file pone.0190830.s001.tif]

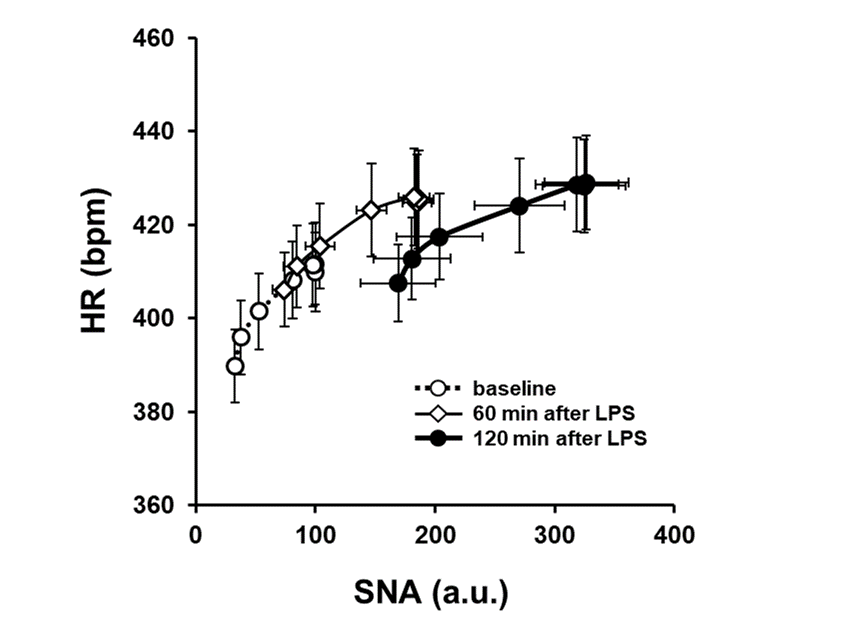

Supplement: S2 Fig — Open-loop static characteristics of sympathetic nerve activity (SNA) and heart rate (HR) obtained at baseline (dotted line with white circles, ○), and 60 min (thin solid line with diamonds, ◇) and 120 min after Lipopolysaccharide (LPS) injection (thick solid line with black circles, ●). Data are expressed as means ± SEM. Data are acquired from the baroreflex open loop condition at baseline and at 60 and 120 min after LPS injection. (TIF) [file pone.0190830.s002.tif]

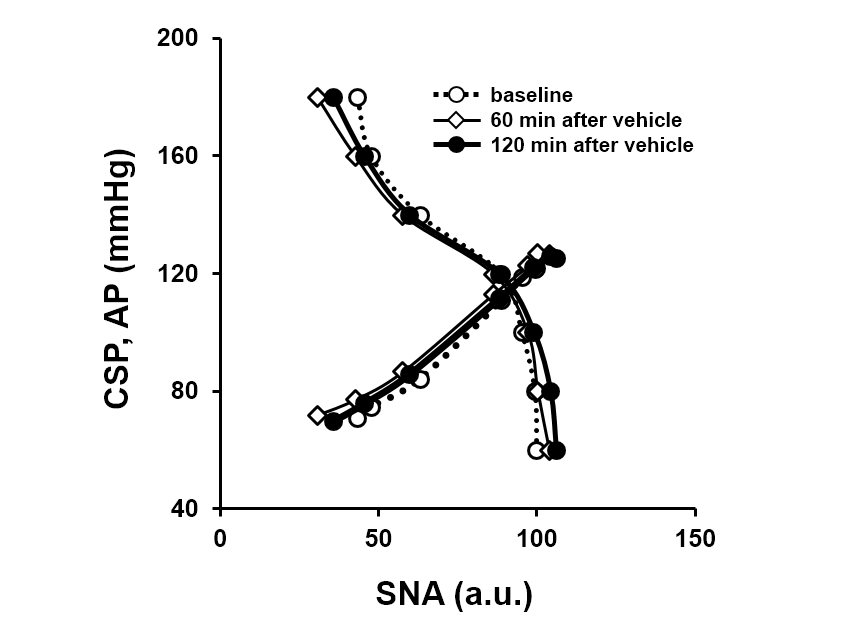

Supplement: S3 Fig — We used saline as vehicle. Methods and protocols for obtaining the baroreflex equilibrium diagram are described in the method section. Averaged baroreflex equilibrium diagrams at baseline (dotted line with white circle, ○), 60 min (thin solid line with diamond, ◇) and 120 min after vehicle injection (thick solid line with black circle, ●) are shown. We did not observe significant changes in the characteristics of central arc or peripheral arc at least until 120 min. Each data was obtained from three rats. (TIF) [file pone.0190830.s003.tif]
